# Direct extraction of topological Zak phase with the synthetic dimension

**DOI:** 10.1038/s41377-023-01126-1

**Published:** 2023-03-29

**Authors:** Guangzhen Li, Luojia Wang, Rui Ye, Yuanlin Zheng, Da-Wei Wang, Xiong-Jun Liu, Avik Dutt, Luqi Yuan, Xianfeng Chen

**Affiliations:** 1 State Key Laboratory of Advanced Optical Communication Systems and Networks, School of Physics and Astronomy, Shanghai Jiao Tong University, Shanghai, 200240 China; 2 Shanghai Research Center for Quantum Sciences, Shanghai, 201315 China; 3 Interdisciplinary Center for Quantum Information and Zhejiang Province Key Laboratory of Quantum Technology and Device, Department of Physics, Zhejiang University, Hangzhou, 310027 China; 4 International Center for Quantum Materials and School of Physics, Peking University, Beijing, 100871 China; 5 International Quantum Academy, Shenzhen, 518048 China; 6 Department of Mechanical Engineering, Institute for Physical Science and Technology, University of Maryland, College Park, MD 20742 USA; 7 Collaborative Innovation Center of Light Manipulation and Applications, Shandong Normal University, Jinan, 250358 China

**Keywords:** Optics and photonics, Optical physics

## Abstract

Measuring topological invariants is an essential task in characterizing topological phases of matter. They are usually obtained from the number of edge states due to the bulk-edge correspondence or from interference since they are integrals of the geometric phases in the energy band. It is commonly believed that the bulk band structures could not be directly used to obtain the topological invariants. Here, we implement the experimental extraction of Zak phase from the bulk band structures of a Su-Schrieffer-Heeger (SSH) model in the synthetic frequency dimension. Such synthetic SSH lattices are constructed in the frequency axis of light, by controlling the coupling strengths between the symmetric and antisymmetric supermodes of two bichromatically driven rings. We measure the transmission spectra and obtain the projection of the time-resolved band structure on lattice sites, where a strong contrast between the non-trivial and trivial topological phases is observed. The topological Zak phase is naturally encoded in the bulk band structures of the synthetic SSH lattices, which can hence be experimentally extracted from the transmission spectra in a fiber-based modulated ring platform using a laser with telecom wavelength. Our method of extracting topological phases from the bulk band structure can be further extended to characterize topological invariants in higher dimensions, while the exhibited trivial and non-trivial transmission spectra from the topological transition may find future applications in optical communications.

## Abstract (web summary)

Experimental extraction of Zak phase from band structures of a Su-Schrieffer-Heeger model in the synthetic frequency dimension, constructed by two coupled ring resonators with bichromatic modulations of different amplitudes.

## Graphical abstract

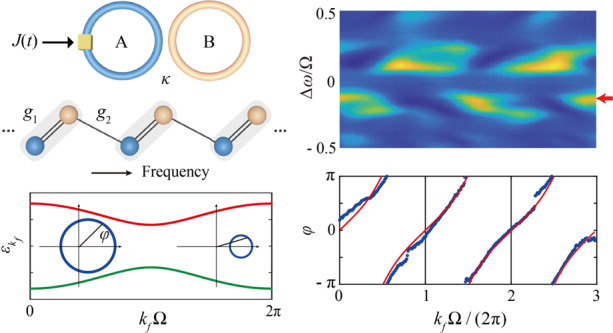

## Introduction

Last few decades have witnessed rapid advances of topological photonic materials with exotic properties, such as topologically protected edge states, unidirectional light transport, high-order topological corner states, topological defects, novel topological phases and phenomena produced in combination with synthetic dimensions, non-equilibrium physics, nonlinearities, non-Hermiticity and quantum effects, which hold important applications in integrated photonic devices^1–13^. The topological phases of matter can be classified by their topological invariants^14–17^. For example, the topology of a one-dimensional (1D) system is characterized by the Zak phase, obtained by integrating the Berry curvature over the first Brillouin zone^18,19^. In the well-known Su-Schriefffer-Heeger (SSH) model^20^, the Zak phase can take two values, which are 0 for the topologically trivial case and *π* for the topologically non-trivial case, corresponding to the winding numbers of ${{{\mathcal{W}}}} = 0$ and ${{{\mathcal{W}}}} = 1$, respectively^21,22^. Probing the Zak phase in 1D photonic systems including the photonic SSH model^23–29^, has been widely demonstrated within several experimental schemes, such as combining Bloch oscillations and Ramsey interferometry^24^, implementing the mean chiral displacement of a particle’s wavepacket^25^, using leaky photonic lattices^26^, and breaking the chiral symmetry in extended SSH models^27,28^. However, due to identical shapes of the bulk band structure in both trivial and non-trivial cases for the SSH lattice, the topological information such as the Zak phase cannot be directly distinguished from the bulk band structure in the current platforms^30^.

Synthetic frequency dimension constructed by coupling the frequency degree of freedom of light has manifested as a powerful platform for creating lattices with artificial connectivities and achieving unusual functionalities that are hard to be achieved in real space^31–38^. Interesting physics associated with the synthetic frequency dimension have been reported by engineering the connectivity through external modulations, such as the Hall ladder with the effective magnetic flux^39^, the dynamic band structure with the off-resonant modulation^40^, non-Hermitian topology with asymmetric spectral hoppings^41,42^, and the flat band with the synthetic stub lattice^43^, where equally-spaced frequency modes are coupled with uniform modulations. However, lattice structures formed by nonuniform connectivities between sites hold richer physics in real space^44–49^, which are only proposed in theory in synthetic space with the frequency dimension, including non-Hermitian SSH lattices^50^ and quadrupole higher-order topological insulators^51^. Therefore, experimental implementation of such unequal couplings between lattice sites is crucial to bring those theoretical proposals into practice, which may also greatly promote the development of synthetic frequency dimension towards constructing more complex lattice structures beyond geometric dimensionality^44–49^.

In this work, we demonstrate the extraction of Zak phase from the bulk band structure of the 1D synthetic SSH model constructed along the frequency dimension of two coupled ring resonators. The symmetric and antisymmetric supermodes in the ring resonator system are connected by the electro-optic phase modulator (EOM), which provides bichromatic sinusoidal modulations at different amplitudes. Such configuration can connect multiple photonic molecules^52^ and then form a 1D SSH model along the frequency axis of light, where the topology is characterized by the Zak phase (0 or *π*). We show that our system possesses its unique feature that the identical shapes of the corresponding band structures under different topological cases can be broken, due to distinct projections of the band structures onto superpositions of the two supermodes. We show that the topological phase information is naturally encoded in the time-resolved projected band structure, which can be extracted from the transmission spectra by choosing the input frequency resonant with the eigenvalue in the momentum space reciprocal to the frequency dimension. Such theoretical proposal is then validated in experiments performed at the telecom wavelength, where measurements in the non-trivial and trivial phases are performed by flexibly reversing the modulation strengths in the bichromatic signal to obtain spectral transmissions. Zak phase values (~ 0 and 0.98*π*) are then extracted in different topological phases. Our scheme to extract Zak phase from the bulk band structure projected onto supermodes holding the phase information is fundamentally different from other systems holding the SSH model^24–28^, and is universal to other topological models^53–57^. Therefore, our work points out a simple route in exploring topological phases of matter with experimental feasibility and reconfigurability in the synthetic frequency dimension, and also holds potential applications in optical communications.

## Results

### Construction of synthetic SSH model

**Fig. 1:**
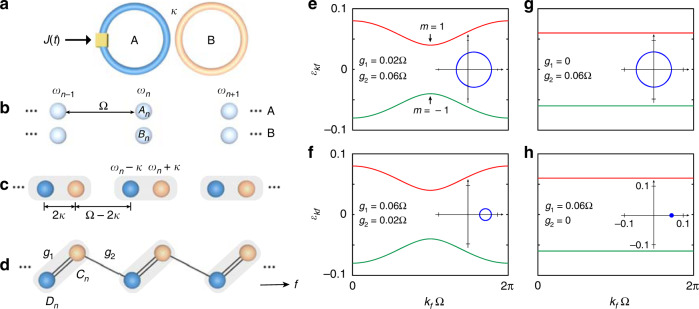
Configuration of the 1D synthetic SSH model. **a** Illustration of two identical coupled ring resonators with coupling strength *κ*, while ring A undergoes dynamic modulation in the form of *J*(*t*) described by Eq. (1). **b** For an individual ring, ring A (B) supports equally-spaced resonant modes labeled as *A*_*n*_ (*B*_*n*_) at frequency *ω*_*n*_. **c** After the effective coupling between two rings, the resonant modes in the system split into two symmetric and antisymmetric supermodes located at *ω*_*n*_ ± *κ*. **d** The discrete supermodes in ring A are coupled by bichromatic modulation *J*(*t*), which can be mapped into a 1D photonic SSH model along the frequency dimension. The supermodes *C*_*n*_ and *D*_*n*_ act as synthetic lattice sites, with alternating hopping amplitudes *g*_1_ and *g*_2_. **e**–**h** The analytical band structures of the synthetic SSH model in (**d**) under different hopping strengths *g*_1_ and *g*_2_. Inserted: the corresponding traces of Re(*G*) and Im(*G*) as the wave vector evolving through the first Brillouin zone

To construct the equivalent SSH model in the synthetic frequency dimension, we start with considering two identical ring resonators labeled as ring A and ring B in Fig. 1(a). In the absence of group-velocity dispersion, ring A (B) supports equally-spaced resonant modes defined as *A*_*n*_ (*B*_*n*_) at frequency *ω*_*n*_ = *ω*_0_ + *n*Ω [see Fig. 1(b)], where *ω*_0_ is the central frequency, *n* is the index of the *n*^th^ mode, and Ω is the free spectral range (FSR) for ring A (B). Modes *A*_*n*_ and *B*_*n*_ at the same *n* can be coupled by the evanescent wave or the fiber coupler between the two rings, with coupling strength *κ*. It then leads to mode splitting with the hybridization of the resonant modes at frequency *ω*_*n*_ into the symmetric supermode *C*_*n*_ at the frequency *ω*_*n*_ + *κ* and the antisymmetric supermode *D*_*n*_ at the frequency *ω*_*n*_ − *κ*^52,58^, which thus constructs unequally spaced synthetic sites in the frequency dimension alternatively separated by Ω_1_ ≡ 2*κ* and Ω_2_ ≡ Ω − 2*κ* as illustrated in Fig. 1(c). An EOM is placed only inside ring A with the external bichromatic signal

1 $$J\left( t \right) = 4g_1\cos \left( {{\Omega}_1t + \phi _1} \right) + 4g_2\cos \left( {{\Omega}_2t + \phi _2} \right)$$

where 4*g*_1_, 4*g*_2_ and *ϕ*_1_, *ϕ*_2_ are the modulation amplitudes and phases. One then obtains the corresponding Hamiltonian of the system in Fig. 1(a) as

2 $$H\left( t \right) = \mathop {\sum}\limits_n {\omega _n\left( {a_n^{\dagger} a_n + b_n^{\dagger} b_n} \right)} + \kappa \mathop {\sum}\limits_n {\left( {a_n^{\dagger} b_n + b_n^{\dagger} a_n} \right)} + \mathop {\sum}\limits_{n,n\prime } {J\left( t \right)a_n^{\dagger} a_{n\prime }}$$

where *a*_*n*_ and *b*_*n*_ ($a_n^{\dagger}$ and $b_n^{\dagger}$) are the annihilation (creation) operators for the modes *A*_*n*_ and *B*_*n*_, respectively. One notes that the last term of Eq. (2) indicates that the applied modulation is off-resonant with resonant modes in ring A. By replacing *a*_*n*_ and *b*_*n*_ with operators $c_n = \left( {a_n + b_n} \right)/\sqrt 2$ and $d_n = \left( {a_n - b_n} \right)/\sqrt 2$, and taking the rotating-wave approximation, one can rewrite the Hamiltonian of Eq. (2) into

3 $$\begin{array}{l}{H_{{{{\mathrm{RWA}}}}} = \mathop {\sum}\limits_n {\left( {\omega _n + \kappa } \right)} c_n^{\dagger} c_n + \mathop {\sum}\limits_n {\left( {\omega _n - \kappa } \right)} d_n^{\dagger} d_n}\\\qquad\quad\;\;\; { + \mathop {\sum}\limits_n {\left[ {g_1e^{ - i\left( {2\kappa t + \phi _1} \right)}c_n^{\dagger} d_n + g_2e^{ - i\left[ {\left( {{\Omega} - 2\kappa } \right)t + \phi _2} \right]}d_n^{\dagger} c_{n - 1} + h.c.} \right]} }\end{array}$$

From Eq. (3), we show the capability of constructing a lattice with nonuniform connectivities between antisymmetric and symmetric supermodes *D*_*n*_ and *C*_*n*_ with alternating intra-cell and inter-cell hopping strengths *g*_1_ and *g*_2_, which is mathematically equivalent with the conventional 1D spatial SSH model but along the frequency axis of light (*f*) as shown in Fig. 1(d). Here, *c*_*n*_ and *d*_*n*_ ($c_n^{\dagger}$ and $d_n^{\dagger}$) denote the annihilation (creation) operators for the supermodes *C*_*n*_ and *D*_*n*_, which hold resonant frequency *ω*_*n*_ + *κ* and *ω*_*n*_ − *κ*, respectively. With definition $\tilde c_n = c_ne^{i\left( {\omega _n + \kappa } \right)t}$and $\tilde d_n = d_ne^{i\left( {\omega _n - \kappa } \right)t}$, Eq. (3) can be transformed to a time-independent Hamiltonian

4 $$H_{{{{\mathrm{RWA}}}},I} = \mathop {\sum}\limits_n {\left( {g_1e^{ - i\phi _1}\tilde c_n^{\dagger} \tilde d_n + g_2e^{ - i\phi _2}\tilde d_n^{\dagger} \tilde c_{n - 1} + h.c.} \right)}$$

We note that the synthetic SSH lattice here in the frequency dimension is infinite if we ignore the gradual change of the FSR and the resulting off-resonance coupling between modes induced by the group velocity dispersion of the fiber^35,59^. Given the fact that there is no hard boundary in the frequency dimension, the infinite synthetic lattice makes the choice of intra-cell and inter-cell hoppings can be arbitrary, and different choices can lead to distinct results. To definitively simulate the topology in a SSH lattice, we therefore strict to the lattice where a pair of supermodes *D*_*n*_ and *C*_*n*_ builds a unit cell, so the connection between *C*_*n*_ and *D*_*n*+1_ is inter-cell. The topology is then well-defined in this physical picture. We emphasize that one can certainly choose the configuration of the SSH lattice other way around, which gives an opposite topology. However, once the SSH configuration is fixed and the unit cell is defined, all the following analysis is consistent and one is possible of extracting the topological invariant in experiments.

We transfer Eq. (4) into the *k*_*f*_ space and obtain the Hamiltonian of the synthetic lattice as $$H_{k_f} = \left( {\begin{array}{*{20}{c}} 0 & G \\ {G^ \ast } & 0 \end{array}} \right)$$, where $G = \left| G \right|e^{i\varphi \left( {k_f} \right)} = g_1e^{ - i\phi _1} + g_2e^{ik_f{\Omega} + i\phi _2}$ and *φ*(*k*_*f*_) = arg(*G*) being the argument of *G*. Here, *k*_*f*_ denotes the wave vector reciprocal to the frequency dimension^33,60^. The topology of the synthetic SSH model can be characterized by the Zak phase following the definition of its spatial counterpart^19^

5 $$\varphi _{{{{\mathrm{Zak}}}}} = \frac{1}{2}{\int}_{ - \pi }^\pi {\frac{{\partial \varphi \left( {k_f} \right)}}{{\partial k_f}}} dk_f$$

The Zak phase takes two values, which are *φ*_Zak_ = *π* for the topologically non-trivial case (*g*_1_ < *g*_2_) and *φ*_Zak_ = 0 for the trivial case (*g*_1_ > *g*_2_). The system degrades to a 1D uniform lattice under the condition *g*_1_ = *g*_2_, which is thus not considered in the following discussion. The corresponding band structures read

6 $$\varepsilon _{k_f,m} = m\sqrt {g_1^2 + g_2^2 + 2g_1g_2\cos \left( {k_f{\Omega} + \phi _1 + \phi _2} \right)}$$

with eigenstates $| {\psi _{k_f,m}} \rangle = ( {\psi _{k_f,m}^C,\,\psi _{k_f,m}^D} )^T = ( {1,\,me^{ - i\varphi }} )^T/\sqrt 2$ and *m* = ±1. $\psi _{k_f,m}^C$ and $\psi _{k_f,m}^D$ are the projections of eigenstates on two supermodes $C_{k_f}$ and $D_{k_f}$ in the *k*_*f*_ space. Note that *φ* is a simple notation for the function *φ*(*k*_*f*_).

Equation (6) indicates that the band structures compose of one upper (*m* = 1) and one lower bands (*m* = −1) as shown in Fig. 1(e)–(h). The trajectories of the real part and imaginary part of *G* as the wave vector sweeping though the first Brillouin zone (*k*_*f*_Ω = 0 → 2*π*) are inserted in Fig. 1(e)–(h), which form circles in the Re(*G*) − Im(*G*) plane, centered at (*g*_1_, 0) with radius *g*_2_. The topology of the system can also be captured by seeing whether the circle passes through the origin, characterized by the winding number (${{{\mathcal{W}}}} = \varphi _{{{{\mathrm{Zak}}}}}/\pi$), where ${{{\mathcal{W}}}} = 0$ denotes the trivial case (*g*_1_ > *g*_2_) and ${{{\mathcal{W}}}} = 1$ denotes the non-trivial case (*g*_1_ < *g*_2_). Equation (6) shows that the band structure is invariant under the exchange of intra-cell and inter-cell coupling strengths *g*_1_ and *g*_2_, although this exchange operation changes the winding number and the Zak phase, moving the lattice from the topologically non-trivial regime to the trivial regime [take Fig. 1(e) and (f) for example]. This invariance or symmetry has made it challenging to distinguish the two topological phases in bulk SSH lattices without looking at edge effects^30^. We will show later how we overcome this challenge by measuring the optical transmission spectra and obtain the time-resolved projected band structure^40,60^, which is the key idea to determine the Zak phase from the bulk band structure in this simulated synthetic SSH lattice.

### Projected band structure from transmission spectra

**Fig. 2:**
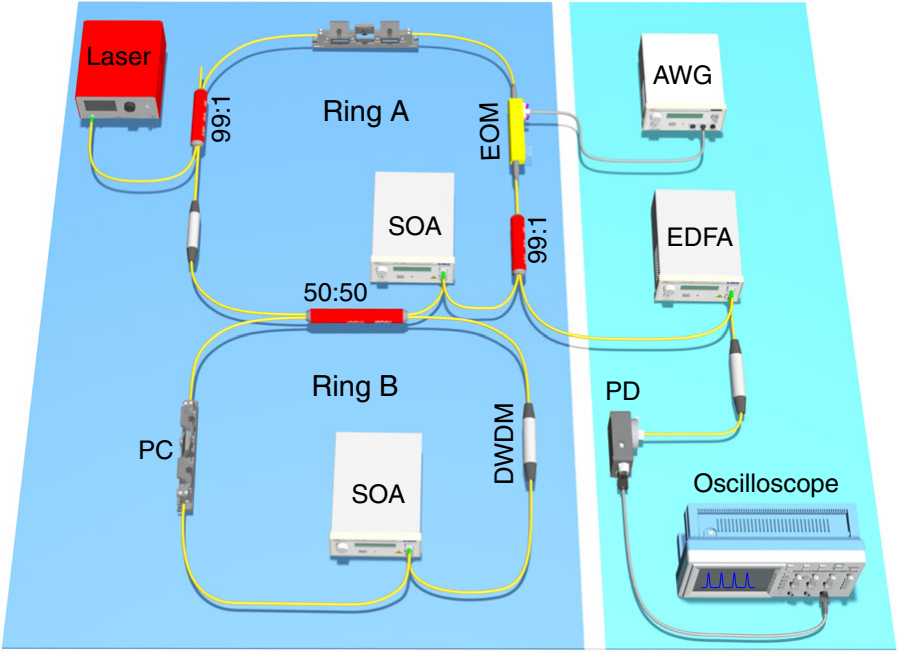
Experimental setup. Ring A and ring B are coupled by a 2 × 2 fiber coupler. EOM: electro-optic phase modulator. SOA: semiconductor optical amplifier. PC: polarization controller. DWDM: dense wavelength division multiplexing. AWG: arbitrary waveform generator. EDFA: erbium-doped optical fiber amplifier. PD: photodiode

To implement our proposal, we use fibers to form two rings in the experiment, which are coupled through a 2 × 2 fiber coupler as shown in Fig. 2 [see Materials and methods]. After calibration, the lengths of both rings are 10.2 m, corresponding to a FSR of *Ω* = 2*πv*_*g*_/*L* = 2*π*·20 MHz, where *v*_*g*_ is the group velocity and *L* is the length of the ring. Without modulation, we observe that the splitting distance of the two supermodes is about 2*π*·6.67 MHz, which gives 2*κ* = Ω/3 and *Ω* − 2*κ* = 2 *Ω*/3 [see Fig. S1 in the Supplementary Information]. To construct the synthetic SSH model, we drive the EOM by a radio frequency (RF) signal in the form of *V*_1_ cos(Ω_1_*t*) + *V*_2_ cos(Ω_2_*t*), where *Ω*_1_ = 2*π*·6.67 MHz, Ω_2_ = 2*π*·13.33 MHz, and *V*_1_, *V*_2_ denote the staggered modulation strengths.

One can obtain the projected band structure of the synthetic lattice following several steps through the time-resolved band structure spectroscopy^40,60^, as we briefly summarize here. First, one collects the drop-port transmission spectrum through the output fiber coupler via linearly scanning the frequency of the input laser source. If the input laser source at frequency *ω* is only detuned around the reference frequency *ω*_0_ + *κ* with detuning ∆*ω*^+*κ*^ ≡ *ω* − (*ω*_0_ + *κ*), one can obtain the output field around the symmetric supermode ($S_{{{{\mathrm{out}}}}}^{ + \kappa }$) as [see Eqs. (S1)–(S9) in the Supplementary Information]

7 $$S_{{{{\mathrm{out}}}}}^{ + \kappa } = - i\frac{{\gamma _{{{\mathrm{A}}}}}}{2}S_{{{{\mathrm{in}}}}}e^{ - i\omega t}\mathop {\sum}\limits_{m = \pm 1} {\left. {\frac{{\psi _{k_f,m}^{C \ast }\left( {\psi _{k_f,m}^C + \psi _{k_f,m}^De^{2i\kappa t}} \right)}}{{{\Delta}\omega ^{ + \kappa } - \varepsilon _{k_f,m} + i\gamma }}} \right|_{k_f = t}}$$

where *S*_in_ is the input laser source, *γ* is the total loss, and *γ*_A_ is the coupling strength between waveguides and ring A. Note that the reference frequency *ω*_0_ (i.e., the 0th resonant mode in a ring) is chosen dependent on the scanning frequency of the input laser source. Similarly, if the input source is detuned around the reference frequency *ω*_0_ − *κ* with detuning ∆*ω*^−*κ*^ ≡ *ω* − (*ω*_0_ − *κ*), the corresponding output filed around the antisymmetric supermode ($S_{{{{\mathrm{out}}}}}^{ - \kappa }$) is

8 $$S_{{{{\mathrm{out}}}}}^{ - \kappa } = - i\frac{{\gamma _{{{\mathrm{A}}}}}}{2}S_{{{{\mathrm{in}}}}}e^{ - i\omega t}\mathop {\sum}\limits_{m = \pm 1} {\left. {\frac{{\psi _{k_f,m}^{D \ast }\left( {\psi _{k_f,m}^Ce^{ - 2i\kappa t} + \psi _{k_f,m}^D} \right)}}{{{\Delta}\omega ^{ - \kappa } - \varepsilon _{k_f,m} + i\gamma }}} \right|_{k_f = t}}$$

The superscripts ±*κ* label the two excited cases around *ω*_0_ ± *κ* separately. Here, the wave vector *k*_*f*_ serves as a time variable^33,60^, due to the discrete translation symmetry along the synthetic frequency dimension. At time *t*, the output field is determined by the eigenvalues and eigenstates at *k*_*f*_ = *t* [see Supplementary Information]. For a fixed detuning Δ*ω*^±*κ*^, the normalized output transmission (${|{S}_{{{{\mathrm{out}}}}}^{ \pm \kappa }/{S}_{\mathrm{in}}|^2}$) has two peaks at ${\Delta}\omega ^{ \pm \kappa } = \varepsilon _{k_f,m}$. Therefore, one can break the transmission spectrum into time slices with time window 2*π*/Ω_1_, and then stack up these time slices as a function of the input frequency detuning ∆*ω*, which reveals the time-resolved band structures of the system. Equations (7) and (8) indicate that if one excites ring A around *ω*_0_ + *κ* and *ω*_0_ − *κ* subsequently by sweeping the input frequency within one free spectral range, two groups of asymmetric projected band structures separated by 2*κ* along the frequency dimension can be acquired, which results from the projections of the band structures onto the superposition of modes $C_{k_f}$ and $D_{k_f}$. Note that each group of projected band structures contains two bands, and the envelope of each band is determined by Eq. (6).

**Fig. 3:**
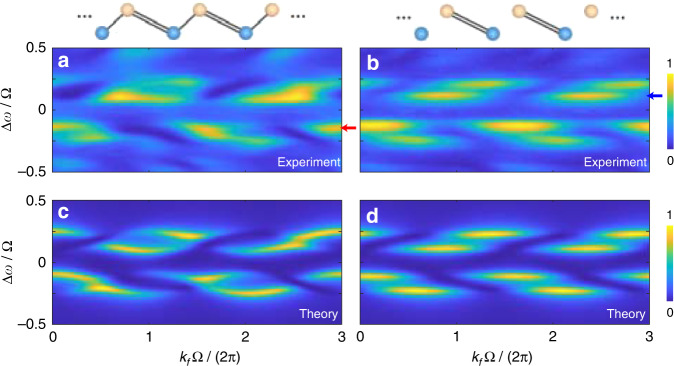
Projected band structures under condition of V_1_ < V_2_. Experimentally observed projected band structures with modulation amplitudes **a** *V*_1_ = 1 V, *V*_2_ = 3 V, and **b** *V*_1_ = 0 V, *V*_2_ = 3 V. Red and blue arrows indicate the chosen bands to apply the Zak phase extraction method. Projected band structures from simulations under coupling strengths **c** *g*_1_ = 0.02 Ω, *g*_2_ = 0.06 Ω, and **d** *g*_1_ = 0, *g*_2_ = 0.06 Ω, with *ϕ*_1_ = *ϕ*_2_ = 0

In the experiment, we consider the condition of *V*_1_ < *V*_2_, and plot the projected band structures in Fig. 3(a), (b). When *V*_1_ = 1 V and *V*_2_ = 3 V are applied [see Fig. 3(a)], two groups of bands are observed at frequency detunings Δ*ω* = Ω/6, while each group contains two dispersive bands with splitting distance determined by modulation strengths. Different from the previous work^40^, here the time window to break the transmission spectrum 2*π*/Ω_1_ equals to three roundtrip time of ring A (3·2*π*/Ω) due to the superposition terms in Eqs. (7) and (8), which gives the periodicity of the projected band structure *k*_*f*_Ω/2*π* ∈ [0, 3]. We also show the projected band structure for the condition with *V*_1_ = 0 V and *V*_2_ = 3 V in Fig. 3(b), corresponding to the special case of *g*_2_/*g*_1_ → ∞ in the SSH model, which causes the four dispersive bands in Fig. 3(a) turning into four flat bands. In addition, we exhibit the simulated bands from Eqs. (7), (8) in Fig. 3(c), (d) under hopping amplitudes *g*_1_ = 0.02 Ω, *g*_2_ = 0.06 Ω and *g*_1_ = 0, *g*_2_ = 0.06 Ω, respectively, which agree with experimental results.

### Zak phase extraction method

Here, we introduce a data-analysis scheme called *resonant method* to extract the important topological phases from the projected band structures in Fig. 3. By further simplifying Eqs. (7), (8) with eigenstates $| {\psi _{k_f,m}}\rangle$, we obtain

9 $$S_{{{{\mathrm{out}}}}}^{ + \kappa } = - i\frac{{\gamma _{{{\mathrm{A}}}}}}{4}S_{{{{\mathrm{in}}}}}e^{ - i\omega t}\mathop {\sum}\limits_{m = \pm 1} {\left. {\frac{{1 + me^{2i\kappa t - i\varphi }}}{{{\Delta}\omega ^{ + \kappa } - \varepsilon _{k_f,m} + i\gamma }}} \right|_{k_f = t}}$$

10 $$S_{{{{\mathrm{out}}}}}^{ - \kappa } = - i\frac{{\gamma _{{{\mathrm{A}}}}}}{4}S_{{{{\mathrm{in}}}}}e^{ - i\omega t}\mathop {\sum}\limits_{m = \pm 1} {\left. {\frac{{1 + me^{ - 2i\kappa t + i\varphi }}}{{{\Delta}\omega ^{ - \kappa } - \varepsilon _{k_f,m} + i\gamma }}} \right|_{k_f = t}}$$

from which one sees that the phase *φ* [*φ*(*k*_*f*_)] of the eigenstates is actually printed in the output field, i.e., the topological phase information being encoded in the time-resolved band structure spectroscopy.

**Fig. 4:**
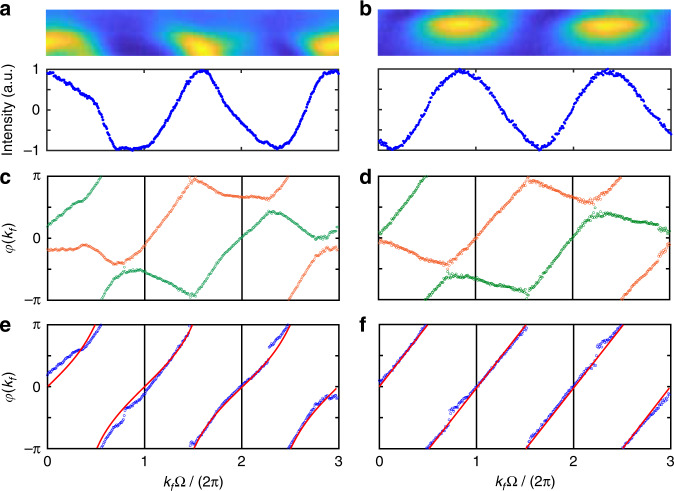
Extraction process of the Zak phase under condition of V1 < V2. **a**, **b** The normalized maximum intensities *S*^2^ of each vertical slice (the lower panels) extracted from the chosen bands (the upper panels) in Fig. 3(a) (red arrow) and Fig. 3(b) (blue arrow), respectively. **c**, **d** The corresponding calculated arguments *φ*(*k*_*f*_) from (**a**) and (**b**) based on Eq. (11). **e**, **f** The selected arguments (blue circles) that satisfy the periodicity of 2*π*/*Ω* from (**c**) and (**d**), in comparison with the theoretical values of arg(*G*) (red lines), respectively

We choose one band from Fig. 3(a) indicated by the red arrow (corresponding to *m* = 1) as an example and re-plot it in Fig. 4(a). By taking the input frequency resonant with the eigenvalue of the chosen band at each *k*_*f*_, one gets ${\Delta}\omega ^{ \pm \kappa } = \varepsilon _{k_f,m}$ to obtain the most significant contribution to the output signal and the phase information from only the chosen band. An intensity parameter $S^2 = 8\gamma ^2\left| {S_{{{{\mathrm{out}}}}}^{ \pm \kappa }} \right|^2/\left( {\gamma _{{{\mathrm{A}}}}^2\left| {S_{{{{\mathrm{in}}}}}} \right|^2} \right) = \left[ {1 + m\cos \left( {2\kappa t - \varphi } \right)} \right]_{k_f = t}$ is defined from Eqs. (9), (10) by minimizing the denominator for the chosen *m* band and neglecting the contribution from the other band, which can be acquired by taking the maximum output intensity value of each vertical slice (fixed *k*_*f*_) from the chosen band. The obtained value *S*^2^ is further normalized to the range of [−1, 1] by using the transform $2S^2/S_{\max }^2 - 1$ as shown in Fig. 4(a), which is then used for decoding the argument *φ*(*k*_*f*_). Following this line, the argument phase *φ*(*k*_*f*_) is deduced as

11 $$\varphi \left( {k_f} \right) = 2\kappa t \pm \arccos \left. {\left[ {m\left( {\frac{{2S^2}}{{S_{\max }^2}} - 1} \right)} \right]} \right|_{k_f = t}$$

with periodicity of *φ*(*k*_*f*_) = *φ*(*k*_*f*_ + 2*π*/Ω) determined by Eq. (6). Both the positive and negative values of the inverse cosine function should be considered, so that one can get the full range of the argument within the range of [−*π*, *π*]. The extracted results are shown in Fig. 4(c), where the orange and green circles represent the acquired pairs of arguments for taking positive and negative inverse cosine values, respectively. Afterwards we select out one argument value from each pair that satisfies the periodicity of *φ*(*k*_*f*_) along the horizontal direction, i.e., 2*π*/Ω, which is shown by the blue circles in Fig. 4(e). We then calculate the Zak phase based on Eq. (5) and acquire $\varphi _{{{{\mathrm{Zak}}}}}^{\exp } \approx 0.98\pi$ (${{{\mathcal{W}}}} \approx 1$). We emphasize that characterizations of the Zak phase from the other three bands in the experiment give the same results. We also demonstrate the Zak phase decoding process for the special case *V*_1_ = 0 V and *V*_2_ = 3 V by choosing a lower band labeled by the blue arrow (*m* = −1) in Fig. 3(b) and show the results in Fig. 4(b), (d) and (f). The flat band leads to linearly varied arguments with wave vector [see Fig. 4(f)] and a Zak phase of $\varphi _{{{{\mathrm{Zak}}}}}^{\exp } \approx 0.975\pi$ (${{{\mathcal{W}}}} \approx 1$). As comparison, we perform calculations on theoretical arguments directly from *G* in Fig. 4(e), (f) (the red lines), which result in *φ*_Zak_ = *π* in both cases. The slight discrepancy between the theoretical and experimental values of the Zak phase is mainly caused by the insufficient accuracy of experimental equipment, the inevitable loss from the system, and the disturbance of the environment, which also cause the extracted phase not exactly periodic in experiments.

**Fig. 5:**
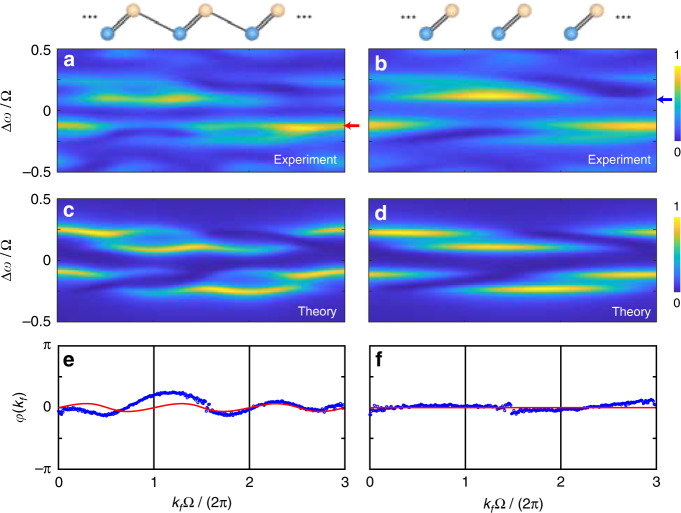
Measurements of the Zak phase under condition of V1 > V2. Experimental observed band structures with modulation amplitudes **a** *V*_1_ = 3 V, *V*_2_ = 1 V, and **b** *V*_1_ = 3 V, *V*_2_ = 0 V. Band structures from simulations under coupling strengths **c** *g*_1_ = 0.06 Ω, *g*_2_ = 0.02 Ω, and **d** *g*_1_ = 0.06 Ω, *g*_2_ = 0, with *ϕ*_1_ = *ϕ*_2_ = 0. **e**, **f** Measured arguments (blue circles) extracted from the chosen bands in (**a**) (red arrow) and (**b**) (blue arrow), compared with theoretical arguments calculated by *G* (red lines), respectively

The topology of a SSH lattice changes from non-trivial to trivial if the ratio between two hopping strengths is flipped. To show this effect, we perform the Zak phase measurement under the condition of *V*_1_ > *V*_2_ corresponding to the trivial case, where projected band structures in experiments are shown in Fig. 5(a) for *V*_1_ = 3 V, *V*_2_ = 1 V and in Fig. 5(b) for *V*_1_ = 3 V, *V*_2_ = 0 V, respectively. The system also exhibits two asymmetric groups of dispersive bands separated by 2*κ* = Ω/3 in Fig. 5(a), which evolves to flat bands in the limit *g*_2_/*g*_1_ → 0 [see Fig. 5(b)]. The corresponding projected band structures from simulations are plotted in Fig. 5(c), (d). One can see a good fit between the simulation results and the experimental measurements. Then, we extract the argument phases from the chosen bands in Fig. 5(a), (b) by following the same procedure in Fig. 4 and show the results in Fig. 5(e), (f) (blue circles), which agree well with the theoretical values calculated from *G* (the red lines). The corresponding Zak phases are integrated as $\varphi _{{{{\mathrm{Zak}}}}}^{\exp } \approx 0.05\pi$ and $\varphi _{{{{\mathrm{Zak}}}}}^{\exp } \approx 0.03\pi$ (${{{\mathcal{W}}}} \approx 0$), respectively, while the theoretical arguments give *ϕ*_Zak_ = 0. Therefore, we show that our system has the capability for directly decoding the topological phase by analyzing the projected band structure from the transmission spectra.

## Discussion

Besides extracting the Zak phase from the bulk band structure, one can also distinguish the topology of the system from the distinct band shapes in the topologically non-trivial case (Fig. 3) and in the trivial case (Fig. 5), which exhibit very different patterns [also see Fig. S2 in the Supplementary Information]. The band shapes in Fig. 3 show characters of shorter segments compared to those in Fig. 5. Unfortunately, the proposed Zak phase extraction method using the intensity parameter *S*^2^ is not applicable for cases with smaller band gap compared to the total loss *γ* including the gapless case at the phase transition point with *V*_1_ = *V*_2_. The key finding of the synthetic SSH model here originates from the frequency difference between the lattice sites in the frequency dimension, which causes the superposition of two supermodes and the topological phase information thus encoded in the bulk band. In addition, the topologically non-trivial and trivial transmission spectra can be converted flexibly by the external modulation, which brings possible ingredient towards exploring spectral non-reciprocity in the future^61–63^, and hence might bring potential opportunity in achieving the active optical isolator and circulator operated at the telecom wavelength^64–66^. Our proposed method for characterizing the topological invariant is universal in the synthetic frequency dimension, and can be used for studying topology with long-range couplings^67,68^. The ability of providing alternating modulations between synthetic lattice sites in experiment therefore offers new possibility to construct more complex lattice structures with nonuniform connectivities in the frequency dimension^44–49^. Our approach in fiber-based ring system can be extended to the microring resonators due to the advance of on-chip integrated photonics^69,70^. Moreover, our results exhibit great potentials in linking towards further connecting photonic molecules^52^, and hence hold applications in photonic computation and quantum systems^71,72^.

In summary, we implement the experimental measurement of topological Zak phase in a synthetic SSH model by utilizing the frequency axis of light, constructed by two coupled ring resonators modulated by bichromatic signals of different amplitudes. We find that the signatures of the topological invariant characterized by the Zak phase are imprinted in the distinguishable time-resolved transmission spectra, which can be extracted by the proposed resonant method. Quantized Zak phases are observed for non-trivial and trivial cases, where experimental measurements show excellent agreement with the results in theory. The main advantage of this method is to extract the topological invariant directly from the bulk band structures. With appropriate designs, the spin or valley degree of freedom is possible to be included in models in the synthetic frequency dimension^37,38^, and hence the corresponding topological invariant may also be directly captured. Our work provides the evidence of directly reading the topological phase from the bulk band structure and paves a new route to explore topological phases in higher-dimensional topological materials^51,73–75^. For example, one way to construct higher-dimensional lattices in the synthetic space is to add long-range connectivities, which can be used to build higher-dimensional synthetic space^76,77^. The superposition of modes along two directions may be obtained so higher-dimensional topological phases can be further studied. We anticipate that future researches in this synthetic frequency platform may find potential applications with physical phenomena in high dimensions and even bulk-defect responses^13^ with reconfigurability, scalability, and flexibility.

## Materials and methods

The two fiber rings are coupled through a 2 × 2 fiber coupler with coupling ratio 50:50. We excite ring A by a tunable laser source (linewidth of 200 kHz) centered at 1550.92 nm, which can be finely scanned over 30 GHz by applying a ramp signal to its frequency modulation input module. A 2 × 2 fiber coupler with coupling ratio 99:1 couples 1% of the laser source to ring A, and a second 99:1 fiber coupler couples out 1% of the signal into the output waveguide for detection. A lithium niobate EOM is placed inside ring A driven by an arbitrary waveform generator (200 MHz bandwidth). The polarization controller is used to calibrate the polarization of resonant frequency modes circling in each ring to the principal axis of EOM. To achieve a high quality factor, a semiconductor optical amplifier is utilized for compensating the losses in the ring, where the amplified spontaneous emission noise is filtered by a dense wavelength division multiplexing centered at 1550.92 nm (international telecommunication union channel 33, 100 GHz bandwidth). The output signal from ring A is amplified by an erbium-doped optical fiber amplifier before being sent to a fast InGaAs photodiode (10 GHz bandwidth) for detection and is then sent to the oscilloscope (5G samples/s with 1 GHz bandwidth) for analysis. To guarantee the two rings having the identical lengths, we also put an unmodulated EOM, and two 2 × 2 fiber couplers with coupling ratio 99:1 in ring B, which are not plotted in Fig. 2.

## Supplementary information

Supplemental Information for Direct extraction of topological Zak phase with the synthetic dimension
