# Supplementary material for: Direct extraction of topological Zak phase with the synthetic dimension
Source: Light Sci Appl. 2023 Mar 29;12:81. doi: 10.1038/s41377-023-01126-1 (PMC10050404; doi:10.1038/s41377-023-01126-1)
Supplement: Supplementary file 1 — Supplemental Information for Direct extraction of topological Zak phase with the synthetic dimension [file 41377_2023_1126_MOESM1_ESM.pdf]

Supplementary Information for: Direct extraction of topological Zak phase with the synthetic dimension

Guangzhen Li,[1,*] Luojia Wang,[1,*] Rui Ye,[1] Yuanlin Zheng,[1,2] Da-Wei Wang,[3] Xiong-Jun Liu,[4,5] Avik Dutt,[6] Luqi Yuan,[1,†] and Xianfeng Chen[1,2,7,‡]

[1]*State Key Laboratory of Advanced Optical Communication Systems and Networks, School of Physics and Astronomy, Shanghai Jiao Tong University, Shanghai 200240, China*

[2]*Shanghai Research Center for Quantum Sciences, Shanghai 201315, China*

[3]*Interdisciplinary Center for Quantum Information and Zhejiang Province Key Laboratory of Quantum Technology and Device, Department of Physics, Zhejiang University, Hangzhou 310027, China*

[4]*International Center for Quantum Materials and School of Physics, Peking University, Beijing 100871, China*

[5]*International Quantum Academy, Shenzhen 518048, China*

[6]*Department of Mechanical Engineering, Institute for Physical Science and Technology, University of Maryland, College Park, MD, 20742, USA*

[7]*Collaborative Innovation Center of Light Manipulation and Applications, Shandong Normal University, Jinan 250358, China*

---

[*]These authors contribute equally to this work.

[†]yuanluqi@sjtu.edu.cn

[‡]xfchen@sjtu.edu.cn

A. Theory of projected band structure measurement in two coupled rings

We extend the technique of time-resolved band structure spectroscopy[1–3] to study the 1D synthetic SSH model constructed in two coupled ring resonators. Consider a two-coupled ring system with the input and output waveguides placed in ring A. The input-output coupled amplitude equations for modal amplitudes of the symmetric and antisymmetric supermodes $v_{c,n}$, $v_{d,n}$ based on the Hamiltonian with the rotating-wave approximation (RWA) in Eq. (3) are given by

$$\begin{aligned}\dot{v}_{c,n} &= \left(-i\omega_n - i\kappa - \gamma\right) v_{c,n} - ig_1 e^{-i2\kappa t - i\phi_1} v_{d,n} - ig_2 e^{i(\Omega - 2\kappa)t + i\phi_2} v_{d,n+1} + i\sqrt{\frac{\gamma_\mathrm{A}}{2}} S_\mathrm{in} e^{-i\omega t} \\ \dot{v}_{d,n} &= \left(-i\omega_n + i\kappa - \gamma\right) v_{d,n} - ig_1 e^{i2\kappa t + i\phi_1} v_{c,n} - ig_2 e^{-i(\Omega - 2\kappa)t - i\phi_2} v_{c,n-1} + i\sqrt{\frac{\gamma_\mathrm{A}}{2}} S_\mathrm{in} e^{-i\omega t} \\ S_\mathrm{out} &= i\sqrt{\frac{\gamma_\mathrm{A}}{2}} \sum_n \left(v_{c,n} + v_{d,n}\right)\end{aligned} \tag{S1}$$

where S_in is the amplitude of the monochromatic input wave with a frequency ω from the input port and S_out is the output field through the drop port of ring A. γ_A is the coupling rate of each mode in ring A coupled to both waveguides and γ is the total decay rate of each mode in the system. The input frequency is slightly detuned from the frequency of the symmetric and antisymmetric supermodes, with definitions $\Delta\omega^{+\kappa} = \omega - (\omega_0 + \kappa)$ and $\Delta\omega^{-\kappa} = \omega - (\omega_0 - \kappa)$, respectively. We then make the transformation

$$\widetilde{v}_{c,n}^{\pm\kappa} = v_{c,n} e^{i\omega_n t + i\kappa t + i\Delta\omega^{\pm\kappa} t} \quad \widetilde{v}_{d,n}^{\pm\kappa} = v_{d,n} e^{i\omega_n t - i\kappa t + i\Delta\omega^{\pm\kappa} t} \tag{S2}$$

to obtain the coupled amplitude equations in the rotated basis as

$$\begin{aligned}\dot{\widetilde{v}}_{c,n}^{\pm\kappa} &= \left(i\Delta\omega^{\pm\kappa} - \gamma\right) \widetilde{v}_{c,n}^{\pm\kappa} - ig_1 e^{-i\phi_1} \widetilde{v}_{d,n}^{\pm\kappa} - ig_2 e^{i\phi_2} \widetilde{v}_{d,n+1}^{\pm\kappa} + i\sqrt{\frac{\gamma_\mathrm{A}}{2}} S_\mathrm{in} e^{i\omega_n t + i\kappa t + i\Delta\omega^{\pm\kappa} t - i\omega t} \\ \dot{\widetilde{v}}_{d,n}^{\pm\kappa} &= \left(i\Delta\omega^{\pm\kappa} - \gamma\right) \widetilde{v}_{d,n}^{\pm\kappa} - ig_1 e^{i\phi_1} \widetilde{v}_{c,n}^{\pm\kappa} - ig_2 e^{-i\phi_2} \widetilde{v}_{c,n-1}^{\pm\kappa} + i\sqrt{\frac{\gamma_\mathrm{A}}{2}} S_\mathrm{in} e^{i\omega_n t - i\kappa t + i\Delta\omega^{\pm\kappa} t - i\omega t}\end{aligned} \tag{S3}$$

Due to the modal translational symmetry along the frequency axis, we can solve equations that are diagonal in the k_f space by defining

$$\Psi_{k_f}^{C,\pm\kappa} = \sum_n \widetilde{v}_{c,n}^{\pm\kappa} e^{-ink_f\Omega} \quad \Psi_{k_f}^{D,\pm\kappa} = \sum_n \widetilde{v}_{d,n}^{\pm\kappa} e^{-ink_f\Omega} \tag{S4}$$

Thus in the k_f space, one gets

$$\begin{aligned}
\dot{\Psi}_{k_f}^{C,\pm\kappa} =&(i\Delta\omega^{\pm\kappa}-\gamma)\Psi_{k_f}^{C,\pm\kappa}-i\left(g_1e^{-i\phi_1}+g_2e^{ik_f\Omega+i\phi_2}\right)\Psi_{k_f}^{D,\pm\kappa}\\
&+i\sqrt{\frac{\gamma_{\mathrm{A}}}{2}}S_{\mathrm{in}}e^{i\kappa t\mp i\kappa t}\sum_n e^{in\Omega\left(t-k_f\right)}\\
\dot{\Psi}_{k_f}^{D,\pm\kappa} =&(i\Delta\omega^{\pm\kappa}-\gamma)\Psi_{k_f}^{D,\pm\kappa}-i\left(g_1e^{i\phi_1}+g_2e^{-ik_f\Omega-i\phi_2}\right)\Psi_{k_f}^{C,\pm\kappa}\\
&+i\sqrt{\frac{\gamma_{\mathrm{A}}}{2}}S_{\mathrm{in}}e^{-i\kappa t\mp i\kappa t}\sum_n e^{in\Omega\left(t-k_f\right)}
\end{aligned} \tag{S5}$$

and $S_{\mathrm{out}}^{\pm\kappa} = i\sqrt{\frac{\gamma_{\mathrm{A}}}{2}}e^{-i\omega t\pm i\kappa t}\left[\Psi_{k_f}^{C,\pm\kappa}(t)\,e^{-i\kappa t}+\Psi_{k_f}^{D,\pm\kappa}(t)\,e^{i\kappa t}\right]\Big|_{k_f=t}$. Using the definition of column vectors $\left|\Psi_{k_f}^{\pm\kappa}\right\rangle = \left(\Psi_{k_f}^{C,\pm\kappa},\Psi_{k_f}^{D,\pm\kappa}\right)^T$ and $\left|S_{\mathrm{in}}^{\pm\kappa}\right\rangle = \left[e^{i(\kappa\mp\kappa)t}\sum_n e^{in\Omega\left(t-k_f\right)}, e^{i(-\kappa\mp\kappa)t}\sum_n e^{in\Omega\left(t-k_f\right)}\right]^T$, we can rewrite Eq. (S5) into a compact form as

$$\left[\Delta\omega^{\pm\kappa}+i\gamma-\left(H_{k_f}-i\partial_t\right)\right]\left|\Psi_{k_f}^{\pm\kappa}\right\rangle+\sqrt{\frac{\gamma_{\mathrm{A}}}{2}}\left|S_{\mathrm{in}}^{\pm\kappa}\right\rangle=0 \tag{S6}$$

where

$$H_{k_f}=\begin{pmatrix} 0 & g_1e^{-i\phi_1}+g_2e^{ik_f\Omega+i\phi_2}\\ g_1e^{i\phi_1}+g_2e^{-ik_f\Omega-i\phi_2} & 0\end{pmatrix} \tag{S7}$$

is the Hamiltonian of the synthetic lattice in the k_f space.

At steady state, $\left|\Psi_{k_f}^{\pm\kappa}(t)\right\rangle$ can be expended by the Floquet eigenstates $\left|\psi_{k_f,m}\right\rangle$ of the system composed of time periodic components with frequencies $2\kappa n_1+(\Omega-2\kappa)\,n_2$ (n_1 and n_2 are arbitrary integers). For the RWA case, eigenstates of H_{k_f} form a complete basis for expending the RWA solution $\left|\Psi_{k_f}^{\pm\kappa}\right\rangle$. The eigenvalues and eigenstates satisfy $H_{k_f}\left|\psi_{k_f,m}\right\rangle = \varepsilon_{k_f,m}\left|\psi_{k_f,m}\right\rangle$, with $\left|\psi_{k_f,m}\right\rangle = \left(\psi_{k_f,m}^{C},\psi_{k_f,m}^{D}\right)^T$ and $m=\pm1$. By taking the inner product of Eq. (S6) with $\left\langle\psi_{k_f,m}\right|$ and only keeping the zero-frequency component in the system, we obtain the expansion coefficients of the output field in terms of the eigenstates as

$$\begin{aligned}
\left\langle\psi_{k_f,m}\middle|\Psi_{k_f}^{+\kappa}\right\rangle &= -\frac{\sqrt{\frac{\gamma_{\mathrm{A}}}{2}}\left\langle\psi_{k_f,m}\middle|S_{\mathrm{in}}^{+\kappa}\right\rangle}{\Delta\omega^{+\kappa}-\varepsilon_{k_f,m}+i\gamma} = -\frac{\sqrt{\frac{\gamma_{\mathrm{A}}}{2}}S_{\mathrm{in}}\psi_{k_f,m}^{C*}}{\Delta\omega^{+\kappa}-\varepsilon_{k_f,m}+i\gamma}\\
\left\langle\psi_{k_f,m}\middle|\Psi_{k_f}^{-\kappa}\right\rangle &= -\frac{\sqrt{\frac{\gamma_{\mathrm{A}}}{2}}\left\langle\psi_{k_f,m}\middle|S_{\mathrm{in}}^{-\kappa}\right\rangle}{\Delta\omega^{-\kappa}-\varepsilon_{k_f,m}+i\gamma} = -\frac{\sqrt{\frac{\gamma_{\mathrm{A}}}{2}}S_{\mathrm{in}}\psi_{k_f,m}^{D*}}{\Delta\omega^{-\kappa}-\varepsilon_{k_f,m}+i\gamma}
\end{aligned} \tag{S8}$$

Thus the output fields from ring A are given by

$$\begin{aligned} S_{\text{out}}^{+\kappa} &= -i\frac{\gamma_{\text{A}}}{2} S_{\text{in}} e^{-i\omega t} \sum_{m=\pm 1} \left. \frac{\psi_{k_f,m}^{C*}\left(\psi_{k_f,m}^{C} + \psi_{k_f,m}^{D} e^{2i\kappa t}\right)}{\Delta\omega^{+\kappa} - \varepsilon_{k_f,m} + i\gamma} \right|_{k_f=t} \\ S_{\text{out}}^{-\kappa} &= -i\frac{\gamma_{\text{A}}}{2} S_{\text{in}} e^{-i\omega t} \sum_{m=\pm 1} \left. \frac{\psi_{k_f,m}^{D*}\left(\psi_{k_f,m}^{C} e^{-2i\kappa t} + \psi_{k_f,m}^{D}\right)}{\Delta\omega^{-\kappa} - \varepsilon_{k_f,m} + i\gamma} \right|_{k_f=t} \end{aligned} \tag{S9}$$

which are Eqs. (7)-(8) in the main text.

B. Supporting figures

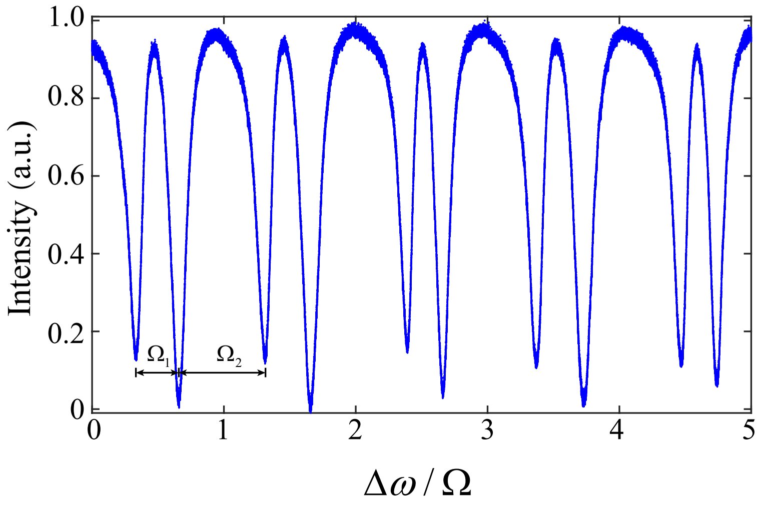

Fig. S1: Measured transmission spectrum from the drop-port output of ring A without modulation, where the horizontal data contains five free spectral ranges.

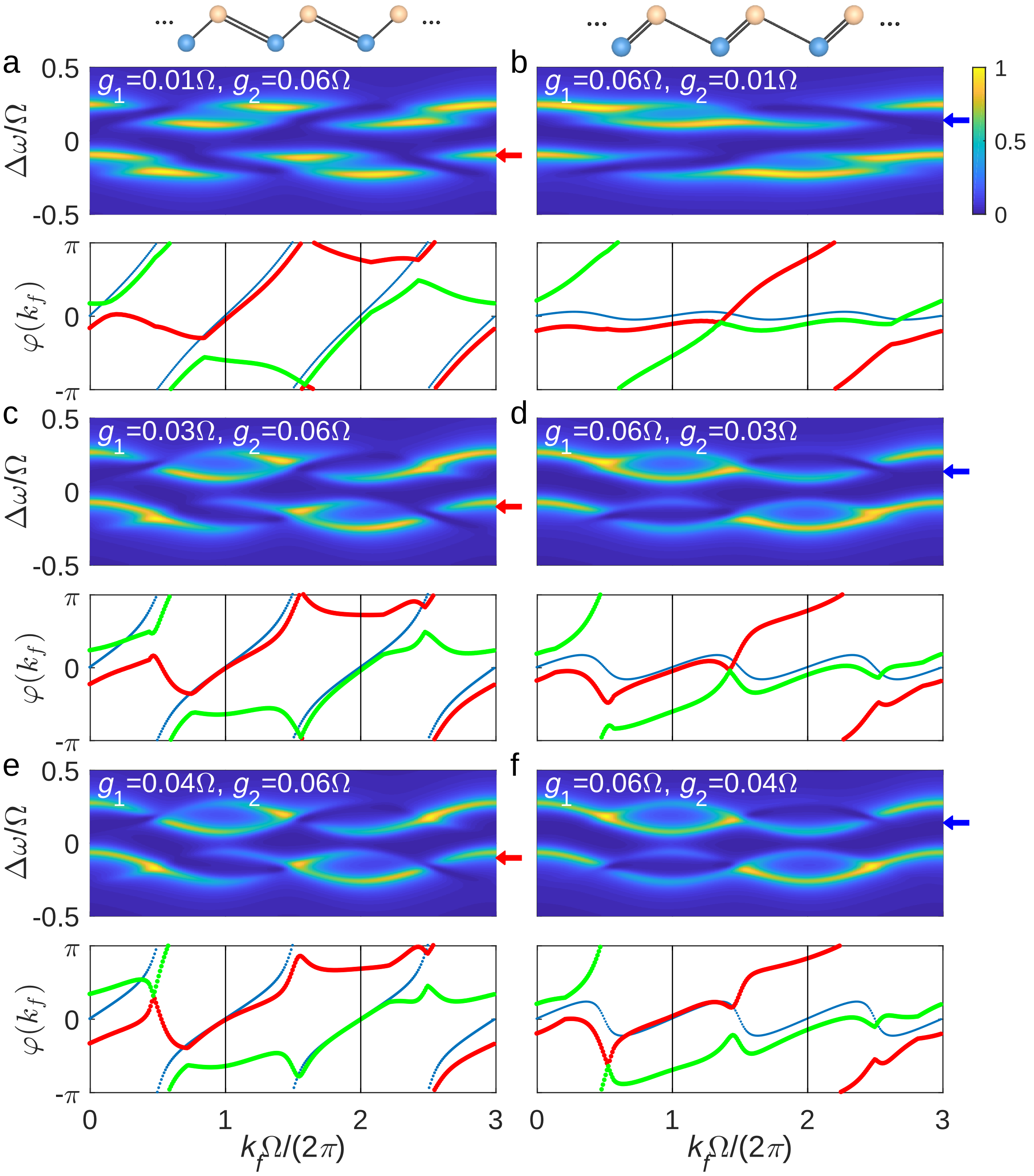

Fig. S2: Projected band structures from simulations and arguments $\varphi(k_f)$ (red and green dots) extracted from the chosen bands indicated by arrows, in comparison with theoretical arguments calculated by G (blue curves). Different coupling strengths of **a** $g_1 = 0.01\Omega$, $g_2 = 0.06\Omega$, **b** $g_1 = 0.06\Omega$, $g_2 = 0.01\Omega$, **c** $g_1 = 0.03\Omega$, $g_2 = 0.06\Omega$, **d** $g_1 = 0.06\Omega$, $g_2 = 0.03\Omega$, **e** $g_1 = 0.04\Omega$, $g_2 = 0.06\Omega$, and **f** $g_1 = 0.06\Omega$, $g_2 = 0.04\Omega$ are taken as comparisons with results in the main text. This proposed resonant method using the intensity parameter S^2 is not applicable when the band gap is close to or smaller than γ as shown in Figs. S2e–S2f. Here $\phi_1 = \phi_2 = 0$ and $\gamma = 0.035\Omega$.

References

1. Dutt, A. *et al*. Experimental band structure spectroscopy along a synthetic dimension. *Nature Communications* **10**, 3122 (2019).
2. Dutt, A. *et al*. A single photonic cavity with two independent physical synthetic dimensions. *Science* **367**, 59--64 (2020).
3. Li, G. Z. *et al*. Observation of flat-band and band transition in the synthetic space. *Advanced Photonics* **4**, 036002 (2022).
